# Depletion of p21-activated kinase 1 up-regulates the immune system of APC^∆14/+^ mice and inhibits intestinal tumorigenesis

**DOI:** 10.1186/s12885-017-3432-0

**Published:** 2017-06-19

**Authors:** Nhi Huynh, Kai Wang, Mildred Yim, Chelsea J. Dumesny, Mauro S. Sandrin, Graham S. Baldwin, Mehrdad Nikfarjam, Hong He

**Affiliations:** Department of Surgery, University of Melbourne, Austin Health, Studley Rd, Heidelberg, VIC 3084 Australia

**Keywords:** PAK1, Intestinal tumour, APC, Lymphocytes

## Abstract

**Background:**

P21-activated kinase 1 (PAK1) stimulates growth and metastasis of colorectal cancer (CRC) through activation of multiple signalling pathways. Up-regulation of CRC stem cell markers by PAK1 also contributes to the resistance of CRC to 5-fluorouracil. The aim of this study was to investigate the effect of PAK1 depletion and inhibition on the immune system and on intestinal tumour formation in APC^∆14/+^ mice.

**Methods:**

The PAK1 KO APC^∆14/+^ mice were generated by cross-breeding of PAK1 KO mice with APC^∆14/+^ mice. Splenic lymphocytes were analysed by flow cytometry, and immunohistochemical staining. The numbers of intestinal tumours were counted. Blood cells were also counted.

**Results:**

Compared to APC^+/+^ mice, the numbers of both T- and B- lymphocytes were reduced in the spleen of APC^∆14/+^ mice. Depletion of PAK1 in APC^∆14/+^ mice increased the numbers of splenic T- and B- lymphocytes and decreased the numbers of intestinal tumours. Treatment of APC^∆14/+^ mice with PF-3758309, a PAK inhibitor reduced the numbers of intestinal tumours and increased the numbers of blood lymphocytes.

**Conclusion:**

Depletion of active PAK1 up-regulates the immune system of APC^∆14/+^ mice and suppresses intestinal tumour development. These observations suggest an important role for PAK1 in the immune response to tumours.

**Electronic supplementary material:**

The online version of this article (doi:10.1186/s12885-017-3432-0) contains supplementary material, which is available to authorized users.

## Background

The threonine/serine kinase P21-activated kinase 1 (PAK1) has been reported to stimulate the growth and/or metastasis of many cancers including those in brain, breast, lung [1], ovarian, prostate, stomach, colon/rectum, liver and pancreas [2, 3]. PAK1 also contributes to therapeutic resistance of cancers of the pancreas [4], colon [5] and lung [1], and thus may become an important target in cancer treatment. We have previously reported that PAK1 stimulates growth and metastasis of colorectal cancer (CRC), through activation of multiple signalling molecules including ERK, AKT [6] and β-catenin [7]. Recently we have also shown that up-regulation of CRC stem cell markers by PAK1 contributes to the resistance of human CRC cell lines to 5-fluorouracil (5-FU) [5]. For example PAK1 activity was increased in 5-FU resistant xenografted CRC tumours with increased expression of stem cell markers, whereas treatment with a PAK1 inhibitor decreased the expression of stem cell markers and sensitized CRC cells to 5-FU [5]. When treated with a PAK1 inhibitor the SCID mice bearing tumour xenografts also had increased size and weight in their spleens. However SCID mice lack functional T cells and B cells (because of defective rearrangement of the T- and B-cell receptor genes) and are therefore immune-compromised [8]. To further investigate the role of PAK1 in the immune response in a tumour-bearing mouse model, an orthotopic model of intestinal cancer in mice with a competent immune system was chosen.

The adenomatous polyposis coli (APC) tumour suppressor gene is mutated in most human CRC [9–11]. Many studies of APC function have been carried out in murine models as the mouse and human APC proteins share 90% amino acid homology [12]. Although the APC^Min/+^ mice has frequently been used to investigate the mechanisms of intestinal tumorigenesis, APC^∆14/+^ mice (which harbour a heterozygous mutation resulting in deletion of exon 14 of the APC tumour suppressor gene [13]) are a better model of human CRC as in addition to the small intestinal tumours observed in APC^Min/+^ mice, APC^∆14/+^ mice also develop tumours in the distal colon and rectum. Tumours isolated from the small intestine and colon/rectum of APC^∆14/+^ mice have greater protein levels of PAK1, β-catenin and hypoxia-inducible factor 1α (HIF-1α) compared to normal intestinal tissue [14]. Furthermore reduction of PAK1 mRNA by siRNA treatment decreased the numbers of small intestinal tumours, and the decrease was associated with reduced protein levels of PAK1, active phospho-PAK1 (pPAK1), β-catenin and HIF-1α [14]. Taken together this evidence indicates an important role for PAK1 in the growth and survival of intestinal tumours in APC^∆14/+^ mice. As the role of PAK1 in the immune response to tumours has not been reported previously, the aim of this study was to investigate the effect of modulation of PAK1 expression and activity on the immune response and the development of intestinal tumours in APC^∆14/+^ mice.

## Methods

All mouse experiments carried out in this paper were approved by the Austin Health Animal Ethics Committee with permit numbers A2010/04016 and A2015/05269.

### APC mice study

PAK1 heterozygous (het) mice in the C57Bl6 strain were bred with either APC^+/+^ or APC^∆14/+^ mice in the same strain to give either APC^+/+^ or APC^∆14/+^ mice on a PAK1 wild type (WT), PAK1 het or PAK1 knockout (KO) background. The APC^∆14/+^ mice on a PAK1 WT, het or KO background were culled at 10 weeks of age, the small intestine and colon/rectum were dissected out, the tumour numbers were counted, and the tumour incidence was calculated by dividing the number of mice that developed tumours by the total number of mice examined and multiplying by 100. Blood was taken for full blood cell counts (Austin Hospital Pathology). The spleens from both APC^+/+^ and APC^∆14/+^ mice on a PAK1 WT, het or KO background were dissected out, weighed and the cells subjected to flow cytometric analysis. The spleens from APC^∆14/+^ mice on a PAK1 WT or KO background were also fixed, and embedded for immunohistochemical (IHC) staining. The tumours and adjacent normal tissues from small intestine and colon/rectum of APC^∆14/+^ mice on a PAK1 WT background were also snap-frozen for Western blot.

Both APC^+/+^ and APC^∆14/+^ mice on a PAK1 WT background were treated with PF-3758309 (25 mg/Kg, dissolved in 5% DMSO in saline) by intra-peritoneal (i.p.) injection from 10 weeks of age. For the first three weeks, the mice were treated with PF-3758309 every other day followed by i.p. twice a week for 7 weeks. This schedule was shown to be effective in a previous study [14]. The mice were culled at 21 weeks of age. The small intestine and colon/rectum were dissected out and the tumour numbers counted. Blood was taken for full blood cell counts. The spleens were dissected, weighed, fixed, and embedded for IHC staining.

### Western blot

Proteins were extracted from tumours and from adjacent normal tissues of small intestine and colon/rectum of APC^∆14/+^ mice using the method described previously [5, 14]. Proteins were separated by running the samples through 10% SDS-PAGE, and then blotted with antibodies against Bmi1 (Gene Tex, Irvine, CA), PAK1 or GAPDH (Cell Signaling, Danvers, MA). Bound antibodies were visualized using ECL reagents (GE Healthcare, Amersham, UK), and the density of each band was analysed using Multi-gauge computer software (Berthold). The correlation of Bmi1 and PAK1 was analysed using the program Sigma Plot 12 (SPSS, Chicago, IL).

### Flow cytometric analysis

Spleens were harvested from 8 to 13 week old mice and homogenised in 0.5% BSA in phosphate-buffered saline (PBS). The splenic cells were collected by centrifugation and red cells were removed with red cell lysis buffer (150 mM NH_4_Cl, 10 mM NaHCO_3_, 0.1 mM EDTA). The remaining cells were further washed in 0.5% BSA in PBS and incubated for 1 h on ice with FITC- or APC-labelled antibodies against B220, CD3, CD8 (1:100) (BD Biosciences, North Ryde, Australia) or CD4 (1:10) (Miltenyi Biotec, Macquarie park, Australia) with respective isotype controls. Cells were washed 3 times in 0.5% BSA in PBS before analysis by FACS Canto II (BD Biosciences). Data were analysed using Weasel software (Cytometry laboratory, Walter and Eliza Hall Institute, Parkville, Australia). Samples from at least 10 mice of each genotype were analysed.

### Immunohistochemical staining

The spleens from APC^∆14/+^ mice in a background of PAK1 WT or PAK KO, or from PF-3758309-treated APC^∆14/+^ mice in a background of PAK1 WT, were paraffin-embedded, sectioned and subjected to IHC staining as described previously [14]. Briefly sample slides were incubated with DAKO peroxidase blocker (Dakopatts, Copenhagen, Denmark) to block endogenous peroxidases. After antigen retrieval by incubation in citrate buffer (10 mM Na_3_citrate), followed by blocking with ultra V block (Thermo Fisher Scientific, Scoresby, Australia), the sample slides were incubated with antibodies against CD3, B220 (Abcam, Melbourne, Australia) or rabbit IgG (Santa Cruze, Dallas, TX) at a 1:100 dilution overnight at 4 °C. After washing with TBST (20 mM Tris, 0.8% NaCl, 0.05% Tween 20, pH 7.6), slides were incubated with horse radish peroxidase-labelled goat anti-rabbit IgG (Dakopatts) for 1 h, followed by diaminobenzidine (Dakopatts) staining. The slides were then dipped in haematoxylin and Scott’s tap water to stain cellular components. Images were taken with a Coolscope (Nikon) and analysed using the Image Pro-Plus 6.0 image analysis program (Media Cybernetics Inc., Silver Spring, MD) or Multi-gauge software.

### Statistical analysis

All values are expressed as means ± standard error. Data were analysed by one-way analysis of variance or t-test as appropriate with the program SigmaStat (SPSS). Differences between two means with *p* < 0.05 were considered significant.

## Results

### PAK1 knockout inhibited intestinal tumours of APC^∆14/+^ mice

**Fig. 1:**
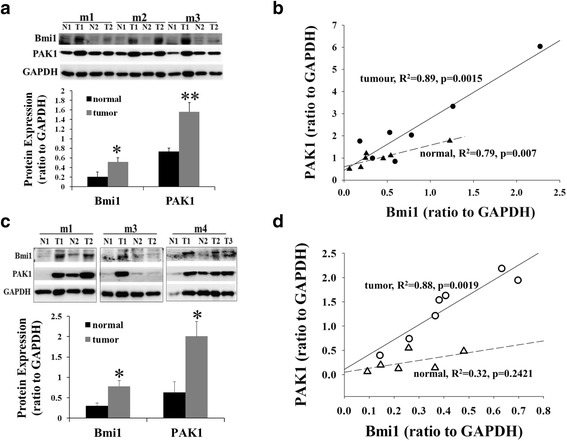
Bmi1 and PAK1 expression are positively correlated in the intestinal tumours of APC^∆14/+^ mice. Both normal (N) and tumour (T) tissues were dissected from colon/rectum (**a**, **b**) or small intestine (**c**, **d**) of APC^∆14/+^ mice. The protein concentrations of Bmi1 and PAK1 were determined by Western blot, normalised to GAPDH, and averaged over 3 replicate blots (**a**, **c**). Regression lines for the correlation of Bmi1 and PAK1 were plotted using the program Sigma Plot 12 (**b**, **d**). R^2^: regression coefficients for normal and tumour samples. m: male, *, *p* < 0.05; **, *p* < 0.01 from samples of tumour tissues compared with samples of adjacent normal tissues

**Fig. 2:**
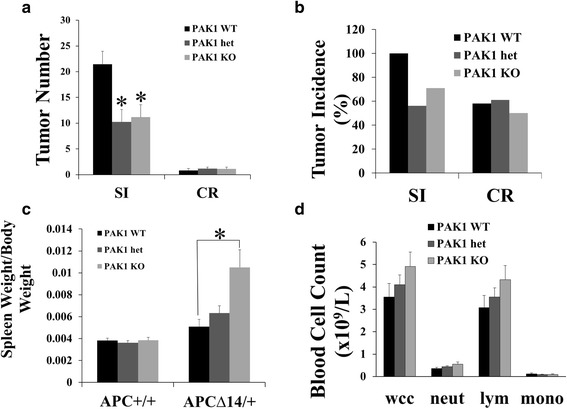
PAK1 knockout inhibited intestinal tumour growth and increased spleen weight of APC^∆14/+^ mice. PAK1 wild type (WT, *n* = 20), heterozygous (het, *n* = 20) and knockout (KO, *n* = 21) APC^∆14/+^ mice were culled at 10 weeks of age. The small intestine and colon/rectum were dissected, and tumour numbers counted (**a**). The tumour incidence was calculated by dividing the number of mice that developed tumours by the total number of mice used for the experiment and multiplying by 100 (**b**). Whole blood was taken for full blood cell count. Samples used in **a**, **b** and **d** were from APC^∆14/+^ mice on a PAK1 WT, het or KO background. Samples used in **c** were from both APC^+/+^ and APC^∆14/+^ mice on PAK1 WT, het or KO backgrounds. *, *p* < 0.05 compared to PAK1 WT APC^∆14/+^ mice

We have previously shown that PAK1 expression and activity were increased, and positively correlated with the levels of HIF-1α and β-catenin, in intestinal tumours in APC^∆14/+^ mice [14]. Here we report that in the tumours of both colon/rectum (Fig. 1a, b) and small intestine (Fig. 1c, d) of APC^∆14/+^ mice, the levels of Bmi1, a well-recognized cancer stem cell marker in intestinal cancer [15–17], were increased. The fact that PAK1 expression was positively correlated with the expression of Bmi1 suggests that PAK1 may play a role in the stem cell development and tumorigenesis of intestinal cancers. This conclusion is strongly supported by the observation that the numbers of tumours in the small intestine of PAK1 heterozygous (het) APC^∆14/+^ and PAK1 knockout (KO) APC^∆14/+^ mice were reduced by over 50% compared to PAK1 wild type (WT) APC^∆14/+^ mice (Fig. 2a). By 10 weeks of age when 100% of mice have tumours in the small intestine, the tumour incidence (i.e. the percentage of the number of mice having tumours in the small intestine (or colon and rectum) in the total number of mice observed) was also decreased in the small intestine of PAK1 het APC^∆14/+^ mice and PAK1 KO APC^∆14/+^ mice (Fig. 2b). PAK1 KO did not affect the numbers of tumours in the colon/rectum (Fig. 2a), where about 50% of PAK1 WT APC^∆14/+^ mice had tumours by 10 weeks of age (Fig. 2b). Taken together these results indicate that PAK1 is required for tumour initiation and progression in murine intestinal mucosa possibly by affecting the stem-like property of these tumours.

### PAK1 knockout up-regulated the immune system of APC^∆14/+^ mice

**Fig. 3:**
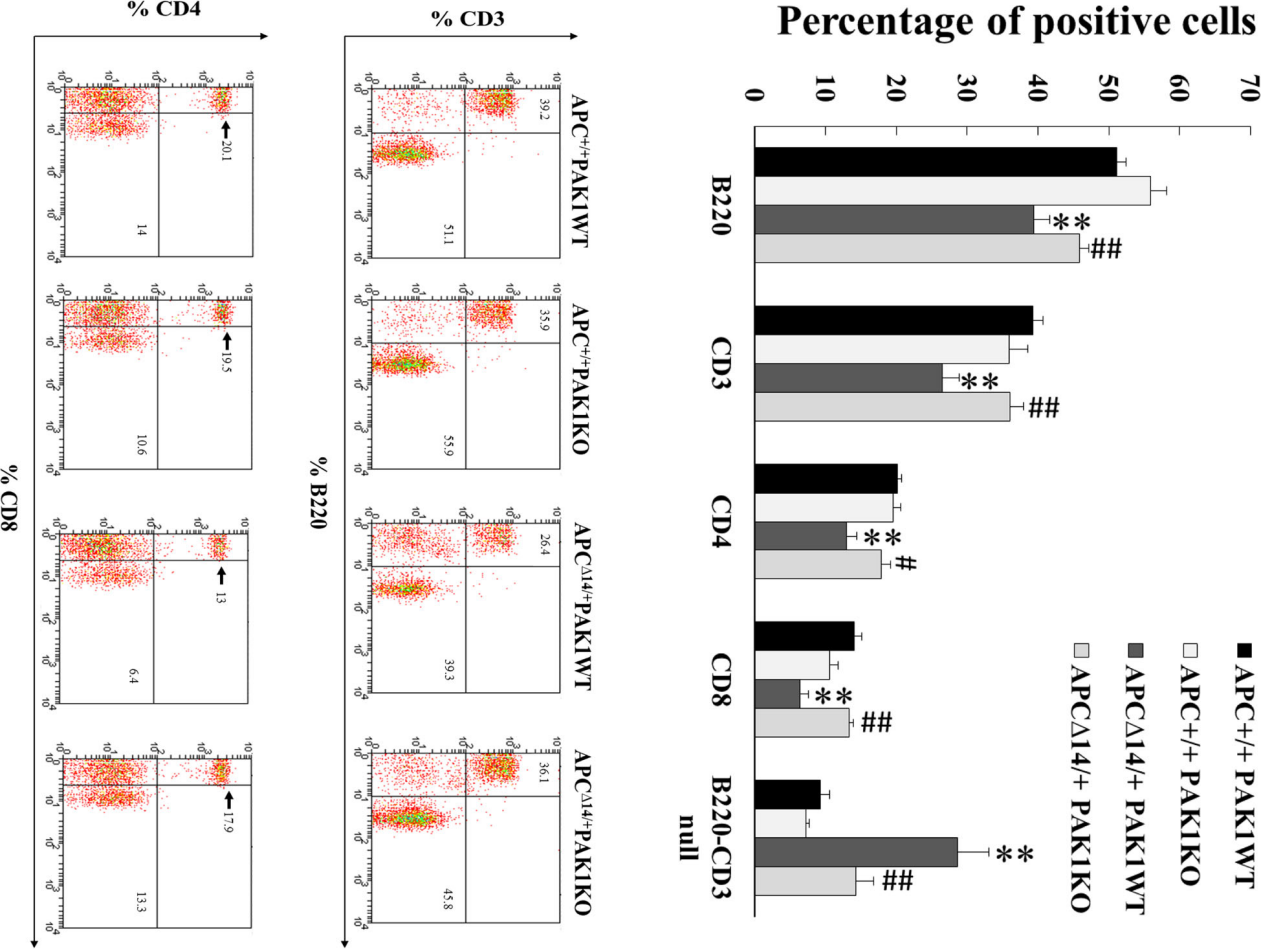
PAK1 knockout increased B- and T-lymphocytes in the spleen of APC^∆14/+^ mice. Fresh spleens were dissected out from 10-week old PAK1 WT APC^+/+^ mice (*n* = 13), PAK1 KO APC^+/+^ mice (*n* = 10), PAK1 WT APC^∆14/+^ mice (*n* = 18) and PAK1 KO APC^∆14/+^ mice (*n* = 16). B220^+^, CD3^+^, CD4^+^ and CD8^+^ lymphocytes were analysed by flow cytometry. The percentage of positive cells was calculated using the Weasel computer program (Cytometry laboratory, WEHI, Parkville). Samples from at least 10 mice of each genotype were analysed **, *p* < 0.01 compared to PAK1 WT APC^+/+^ mice. #, *p* < 0.05, ##, *p* < 0.01 compared to PAK1 WT APC^∆14/+^ mice

**Fig. 4:**
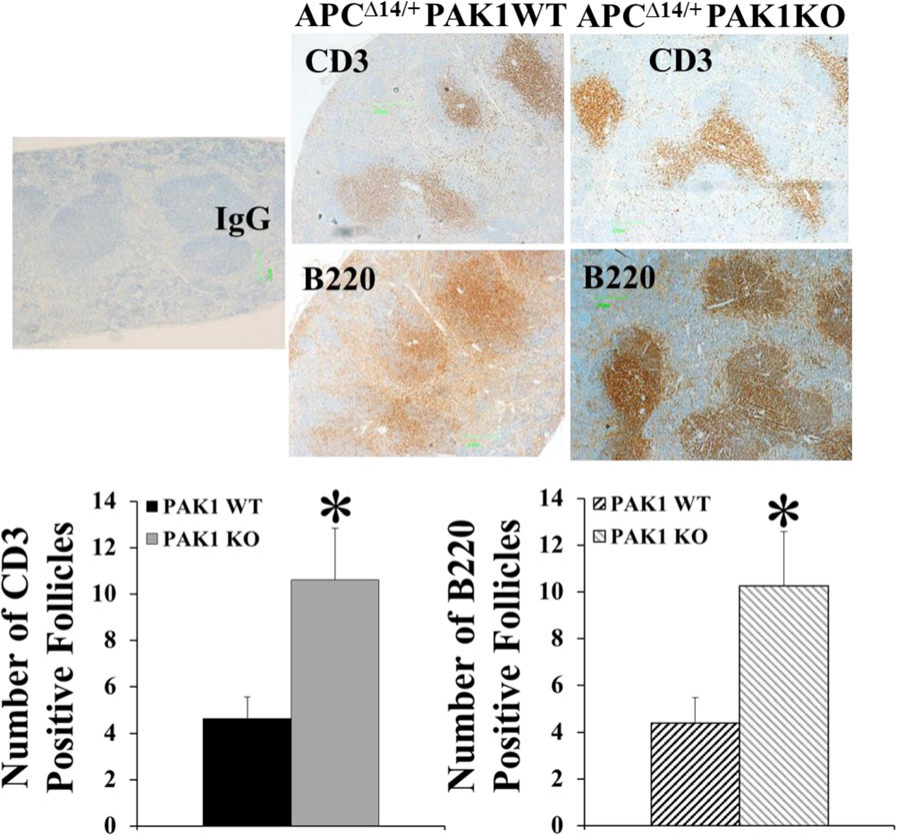
PAK1 knockout increased the numbers of CD3^+^ and B220^+^ follicles in the spleen of APC^∆14/+^ mice. The spleens of with PAK1 WT APC^∆14/+^ mice (*n* = 10) or PAK1 KO APC^∆14/+^ mice (*n* = 12) were immunohistochemically stained with antibodies against CD3 or B220, and numbers of positively stained follicles were counted. *, *p* < 0.05 compared to PAK1 WT APC^∆14/+^ mice

The spleen weight of PAK1 KO APC^∆14/+^ mice was significantly increased compared to PAK1 WT APC^∆14/+^ mice (Fig. 2c). To compare the splenic lymphocytes of PAK1 WT APC^∆14/+^ and PAK1 KO APC^∆14/+^ mice, spleen cells were analysed by flow cytometry, and the percentages of B220^+^ B cells, and CD3^+^, CD4^+^ or CD8^+^ T cells, were calculated. Within APC wild type (APC^+/+^) background, there was no difference in the lymphocytes inspected between PAK1 WT and KO mice (Fig. 3). The percentage of B220^+^ B cells, and of CD3^+^, CD4^+^ and CD8^+^ T cells, were significantly decreased in PAK1 WT APC^∆14/+^ mice compared to PAK1 WT APC^+/+^ mice (Fig. 3). In contrast the percentage of B220^+^ B cells, and of CD3^+^, CD4^+^ and CD8^+^ T cells in PAK1 KO APC^∆14/+^ mice, were similar to those in PAK1 KO APC^+/+^mice and PAK1 WT APC^+/+^ mice (Fig. 3). In other words, the APC^∆14^ mutation decreased all populations of splenic lymphocytes, but PAK1 knockout in APC^∆14/+^ mice reversed this effect, so that all populations of splenic lymphocytes in PAK1 KO APC^∆14/+^ mice were similar to PAK1 WT APC^+/+^ mice. Consistent with these results, immunohistochemical staining showed that the numbers of CD3^+^ and B220^+^ follicles in the spleens of PAK1 KO APC^∆14/+^ mice were significantly increased compared to PAK1 WT APC^∆14/+^ mice (Fig. 4). However differences in the blood white cell and lymphocyte counts between PAK1 KO APC^∆14/+^ mice and PAK1 WT APC^∆14/+^ mice did not reach statistical significance (Fig. 2d). Together these data show that both B- and T- lymphocytes in the spleen of PAK1 KO APC^∆14/+^ mice were significantly increased. These observations indicate that PAK1 KO may stimulate the immune system of APC^∆14/+^ mice by up-regulation of splenic lymphocytes, and suggest that PAK1 may play a role in the immune response to intestinal tumours.

### PF-3758309 inhibited intestinal tumour growth and increased blood lymphocytes and neutrophils in APC^∆14/+^ mice

**Fig. 5:**
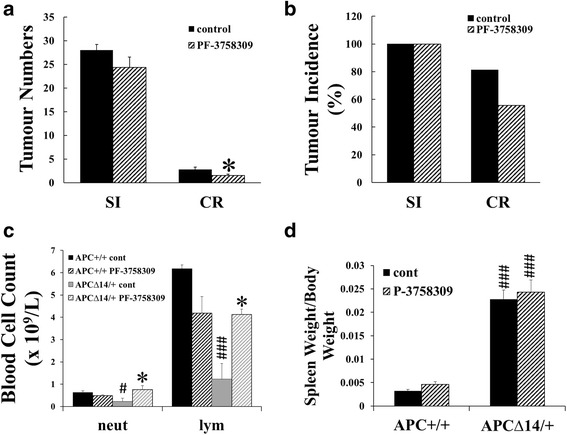
PF-3758309 inhibited colorectal tumour growth and increased the numbers of circulating neutrophils and lymphocytes in APC^∆14/+^ mice. 10-week old APC^+/+^ (*n* = 6) and APC^∆14/+^ (*n* = 18) mice were treated with PF-3758309 by peritoneal injection (25 mg/Kg) for 10 weeks. Control APC^+/+^ (*n* = 6) and APC^∆14/+^ (*n* = 16) mice were treated with 5% DMSO in saline. The mice were culled by 21 weeks of age, blood was taken for full blood cell count (**c**), small intestine and colon/rectum were dissected out and tumours counted (**a**), and the spleens were weighed (**d**). The tumour incidence (**b**) was calculated by dividing the number of mice that developed tumours by the total number of mice used for the experiment and multiplying by 100. *, *p* < 0.05 compared to control; ###, *p* < 0.001 compared to APC^+/+^ mice

To determine the effect of PF-3758309 (selective for PAK1 and PAK4 [5, 18]) on the immune response to intestinal tumour growth in mice with a functional immune system, APC^∆14/+^ mice were treated with PF-3758309 from 10 to 20 weeks of age. Tumour numbers in the small intestine, and colon and rectum, were compared, and spleen weight and the numbers of lymphocytes were measured. PF-3758309 significantly reduced the numbers of tumours in the colon and rectum, but not in the small intestine (Fig. 5a&b), probably due to the fact that the numbers of tumours in small intestine have already been established by 10 weeks of age, and therefore could not be affected by later treatment with PF-3758309. By 21 weeks of age, the numbers of blood lymphocytes and neutrophils were decreased in APC^∆14/+^ mice compared to APC^+/+^ mice; PF-3758309 treatment significantly increased the numbers of blood lymphocytes in APC^∆14/+^ mice, but did not affect the numbers of lymphocytes in the blood of APC^+/+^ mice (Fig. 5c). Similarly PF-3758309 treatment increased the numbers of blood neutrophils in APC^∆14/+^ mice without significantly affecting the numbers of blood neutrophils in APC^+/+^ mice (Fig. 5c). Together these results indicated that PF-37583009 inhibited the growth of colorectal tumours, and the inhibition was associated with an increase in the numbers of blood neutrophils and lymphocytes.

By 21 weeks of age, APC^∆14/+^ mice developed enlarged spleens with the increase in size and weight probably a compensatory response to anaemia. PF-3758309 treatment did not affect the spleen weight and size of either APC^∆14/+^ mice or APC^+/+^ mice (Fig. 5d). No significant changes were observed in either CD3^+^ or B220^+^ follicles with or without PF-3758309 treatment (data not shown).

## Discussion

Our recent report, that the PAK inhibitor PF-3758309 suppressed xenograft CRC cell growth in SCID mice by inhibition of PAK1 activity [5], suggested that PAK1 is the target of PF-3758309 that mediates its inhibitory effect on colorectal cancer growth. The observation that the treated mice also had increased numbers of white blood cells and lymphocytes, as well as increased spleen size, weight and white pulp area (Additional file 1: Figure S1), suggested that PAK1 might affect the immune response to CRC, and prompted us to determine the effect of inhibition or depletion of PAK1 on the immune response to tumour development in an orthotopic intestinal tumour model with a functional immune system. Treatment of APC^∆14/+^ mice with PF-3758309 for 10 weeks increased the numbers of circulating neutrophils and lymphocytes and reduced the numbers of tumours in the colon and rectum. However the effect of PF-3758309 on splenic lymphocytes in APC^∆14/+^ mice was complicated by the fact that untreated mice developed splenomegaly by 20 weeks of age in response to anaemia, presumably caused by intestinal bleeding (data not shown). Splenomegaly was also observed in untreated 20-week old PAK1 KO APC^∆14/+^ mice. Since the 10-week old APC^∆14/+^ mice had spleens of normal size and weight compared to APC^+/+^ mice, we chose to determine the effect of PAK1 KO on intestinal tumour development and splenic lymphocytes in 10-week old mice. Our results demonstrate for the first time that PAK1 depletion up-regulated the immune system while inhibiting intestinal tumour initiation and progression in APC^∆14/+^ mice. The fact that PAK1 depletion by gene knockout did not affect the immune system of APC^+/+^ mice, but up-regulated the immune system by increasing the splenic weight and numbers of lymphocytes of APC^∆14/+^ mice, indicated that the up-regulation of the immune system by PAK1 depletion may be related to tumours induced by APC gene mutation.

The spleen, as an important secondary lymphoid organ, regulates the immune response by specific cell movement and contact, and by affecting chemokine secretion [19]. In tumour-bearing mice, an increased number of B cells in the spleen is associated with cancer regression and the presence of B220^+^/CD86^+^ activated antigen-presenting B cells in the lymphoid organs and in the periphery [20]. In an orthotopic murine model of pancreatic cancer, transferring the splenic T cells after short-term blockade of PD1 (programed cell death protein 1) inhibited tumour progression and extended the survival of non-treated tumour-bearing recipient mice [21]. These reports have shown that both B and T cells from the spleen play important roles in the immune response to tumours. In agreement with these findings, knockout of PAK1 in the intestinal tumour model of APC^∆14/+^ mice inhibited intestinal tumour initiation and progression and increased the numbers of both B and T cells in the spleen, although no significant difference was observed in the numbers of circulating neutrophils and lymphocytes. Furthermore inhibition of PAK1 by treatment of APC^∆14/+^ mice with PF-3758309 suppressed tumour growth in the colon and rectum and increased the numbers of neutrophils and lymphocytes in the blood. Taken together our findings indicate that inhibition of PAK1 not only supresses intestinal tumour progression, but also stimulates the immune response by up-regulation of immune system.

Inhibition of PAK1 seems to target the initial development of tumours more than their later growth. By 10 weeks of age APC^∆14/+^ mice had developed significant numbers of tumours in the small intestine, with much lower numbers in the colon and rectum (Fig. 2a). When comparing 10-week old and 20-week old APC^∆14/+^ mice, the fact that there was no difference in either the number of tumours (Fig. 2a vs Fig. 5a) or tumour incidence (Fig. 2b vs Fig. 5b) in the small intestine indicated that the initiation of tumours in the small intestine was established by 10 weeks, and that from 10 to 20 weeks tumours grew bigger rather than increasing in number. PAK1 knockout decreased both the number of tumours and tumour incidence in the small intestine of 10-week old APC^∆14/+^ mice (Fig. 2b). In contrast PF-3758309 treatment, which was only started when mice had reached 10 weeks of age, did not affect the numbers of pre-existing tumours in the small intestine (Fig. 5a). The observation that PAK1 knockout did not have a significant effect on the number of tumours or tumour incidence in the colon and rectum of 10-week old APC^∆14/+^ mice is perhaps due to the lower numbers and slower development of tumours in the colon and rectum of these mice. By 20 weeks of age 80% of APC^∆14/+^ mice had developed tumours in the colon and rectum (Fig. 5b), and PF-3758309 treatment significantly reduced the number of tumours in the colon and rectum (Fig. 5a) and also reduced the tumour incidence in the colon and rectum to under 60% (Fig. 5b). The possibility that PF-3758309 treatment may have affected the size of pre-existing tumours will be the subject of further investigation. Taken together the data obtained from either PAK1 KO APC^∆14/+^ mice or PF-3758309-treated APC^∆14/+^ mice imply a more important role for PAK1 in the initiation of intestinal tumours rather than their subsequent growth.

The observations that PAK1 KO inhibited intestinal tumour progression and up-regulated the immune system by increasing the numbers of the splenic lymphocytes in APC^∆14/+^ mice, but that PAK1 KO did not affect the numbers of splenic lymphocytes in APC^+/+^ mice, indicate a possible role of PAK1 in the immune response to tumours induced by mutation in the APC gene. There have been very few previous reports about the role of PAK in the immune system. In virus infections, the majority of interactions of PAKs promote virus replication, spread, and/or immune evasion [22], although there is also an antiviral role of PAKs. PAK1 and PAK2 are involved in the inhibition of T cell development/migration, T cell receptor signalling and the B cell immunoglobulin isotype switch in response to human immunodeficiency virus (HIV) infection [22]. These observations indicate that PAKs enhance virus infection in part by inhibition of the immune response, and therefore suppression of PAKs would be expected to inhibit virus infection and up-regulate the immune system. In agreement with the above report, the data presented here demonstrate that reduction of active PAK1 by gene knockout or by PF-3758309 treatment suppressed intestinal tumour development and up-regulated the immune system in the orthotopic intestinal cancer model of APC^∆14/+^ mice. Although PAK1 is well known for its role in cancer development, our data has highlighted for the first time a role for PAK1 in the immune response to tumours.

Compared to APC^+/+^ mice, the immune system of APC^∆14/+^ mice is down-regulated with decreased numbers of splenic lymphocytes. A progressive loss of immature and mature T cells and of immature B cells and B progenitor cells in bone marrow, and splenic natural killer cells, has been reported previously in APC^Min/+^ mice [23]. Loss of APC function affects thymocyte development via β-catenin stabilization and chromosome segregation [24]. Our observation that depletion of PAK1 up-regulated the immune system by increasing the numbers of splenic lymphocytes of APC^∆14/+^ mice, but did not affect the immune system of APC^+/+^ mice, suggests a role for PAK1 in the down-regulation of the immune system that occurred to APC^∆14/+^ mice, although the mechanism remains to be investigated. As mentioned above, PAK1 promotes the inhibition of immune system induced by HIV infection [22]. Taken together these separate lines of evidence indicate that PAK1 may promote inhibition and/or a deficiency in the immune system caused by virus infection or other conditions. Thus inhibition of PAK1 might up-regulate the immune system and reverse the defect.

## Conclusions

Depletion of PAK1 up-regulated the immune system and inhibited intestinal tumorigenesis in APC^∆14/+^ mice. PAK1 appears to be involved in the inhibition and/or down-regulation of the immune system in response to tumour development. Thus inhibition of PAK1 should reverse the suppression and/or deficiency in the immune system, and hence up-regulate the immune system to promote a stronger immune response in disease settings such as cancer.

## Additional file

Additional file 1: Figure S1. PF-3758309 increased the numbers of white blood cells, and splenic weight and white pulp area, in SCID mice. HCT116 human CRC cells were grown as xenografted tumours in SCID mice for 2 weeks. The mice (*n* = 6) were then treated with PF-3758309 by peritoneal injection (25 mg/Kg) for a further 2 weeks. The mice were then culled, the blood taken for blood cell count and the spleens weighed, fixed in formalin, sectioned and stained with H&E. Control mice (*n* = 4) were treated with 5% DMSO in saline. Cont: control; WCC: white blood cell; neut: neutrophil; lym: lymphocyte; mono: monocytes. *, *p* < 0.05, **, *p* < 0.01, compared to control (PPTX 566 kb)

## Abbreviations

5-FU: 5-fluorouracil
APC: Adenomatous polyposis coli
CRC: Colorectal cancer
HIF-1α: Hypoxia-inducible factor 1α
PAK1: P21-activated kinase 1
