# Supplementary figures and images for: Depletion of p21-activated kinase 1 up-regulates the immune system of APC∆14/+ mice and inhibits intestinal tumorigenesis
Source: BMC Cancer. 2017 Jun 19;17:431. doi: 10.1186/s12885-017-3432-0 (PMC5477105; doi:10.1186/s12885-017-3432-0)

## Slide 1
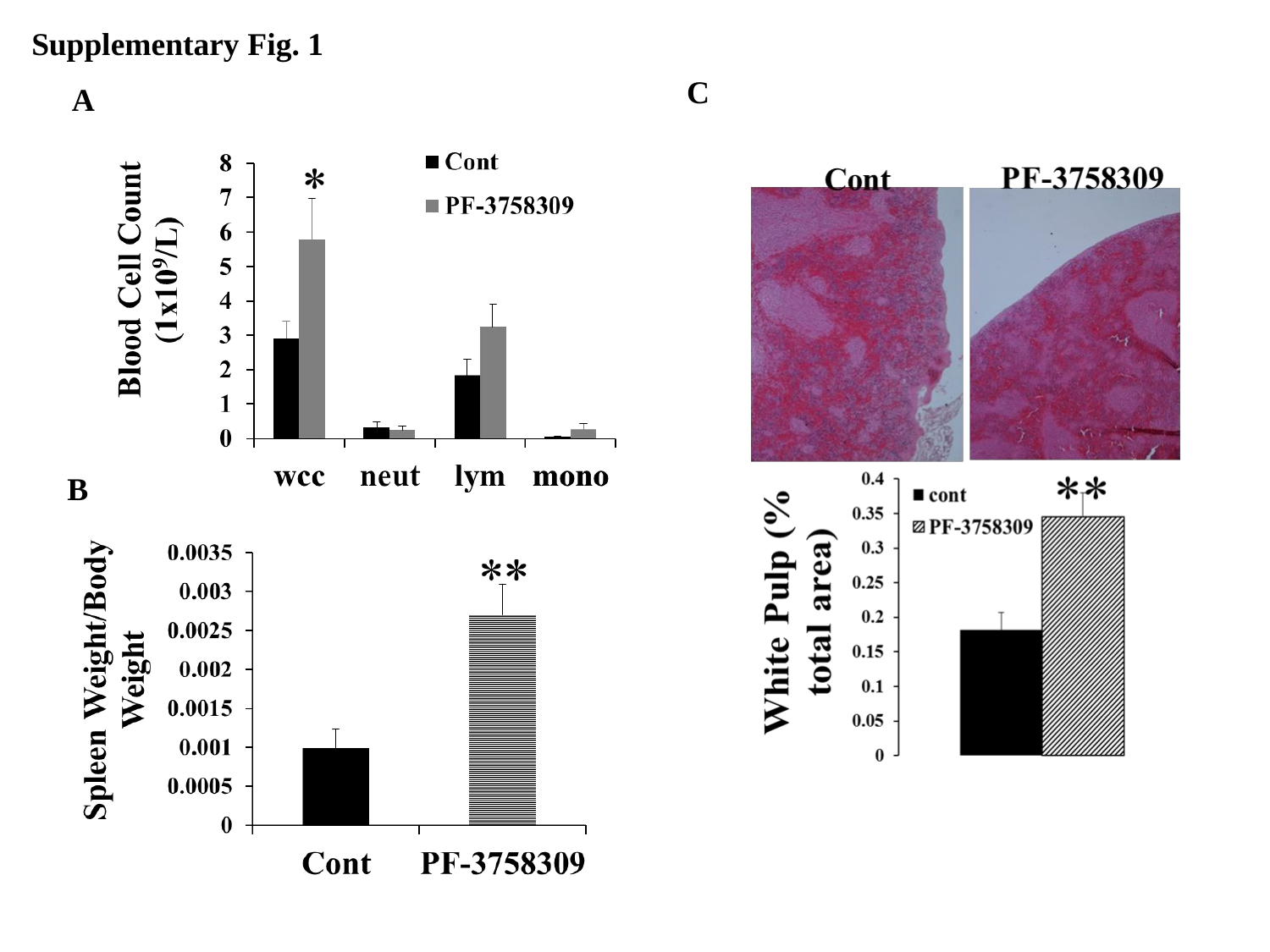

Supplementary Fig. 1
C
A
B

Supplement: Additional file 1: Figure S1. — PF-3758309 increased the numbers of white blood cells, and splenic weight and white pulp area, in SCID mice. HCT116 human CRC cells were grown as xenografted tumours in SCID mice for 2 weeks. The mice (n = 6) were then treated with PF-3758309 by peritoneal injection (25 mg/Kg) for a further 2 weeks. The mice were then culled, the blood taken for blood cell count and the spleens weighed, fixed in formalin, sectioned and stained with H&E. Control mice (n = 4) were treated with 5% DMSO in saline. Cont: control; WCC: white blood cell; neut: neutrophil; lym: lymphocyte; mono: monocytes. *, p < 0.05, **, p < 0.01, compared to control (PPTX 566 kb) [file 12885_2017_3432_MOESM1_ESM.pptx]
